# Supplementary material for: A lipopolysaccharide‐dependent phage infects a pseudomonad phytopathogen and can evolve to evade phage resistance
Source: Environ Microbiol. 2022 Aug 1;24(10):4834–52. doi: 10.1111/1462-2920.16106 (PMC9796965; doi:10.1111/1462-2920.16106)
Supplement: Supplementary file 1 — APPENDIX S1 Supplementary information. [file EMI-24-4834-s001.pdf]

Supplementary data

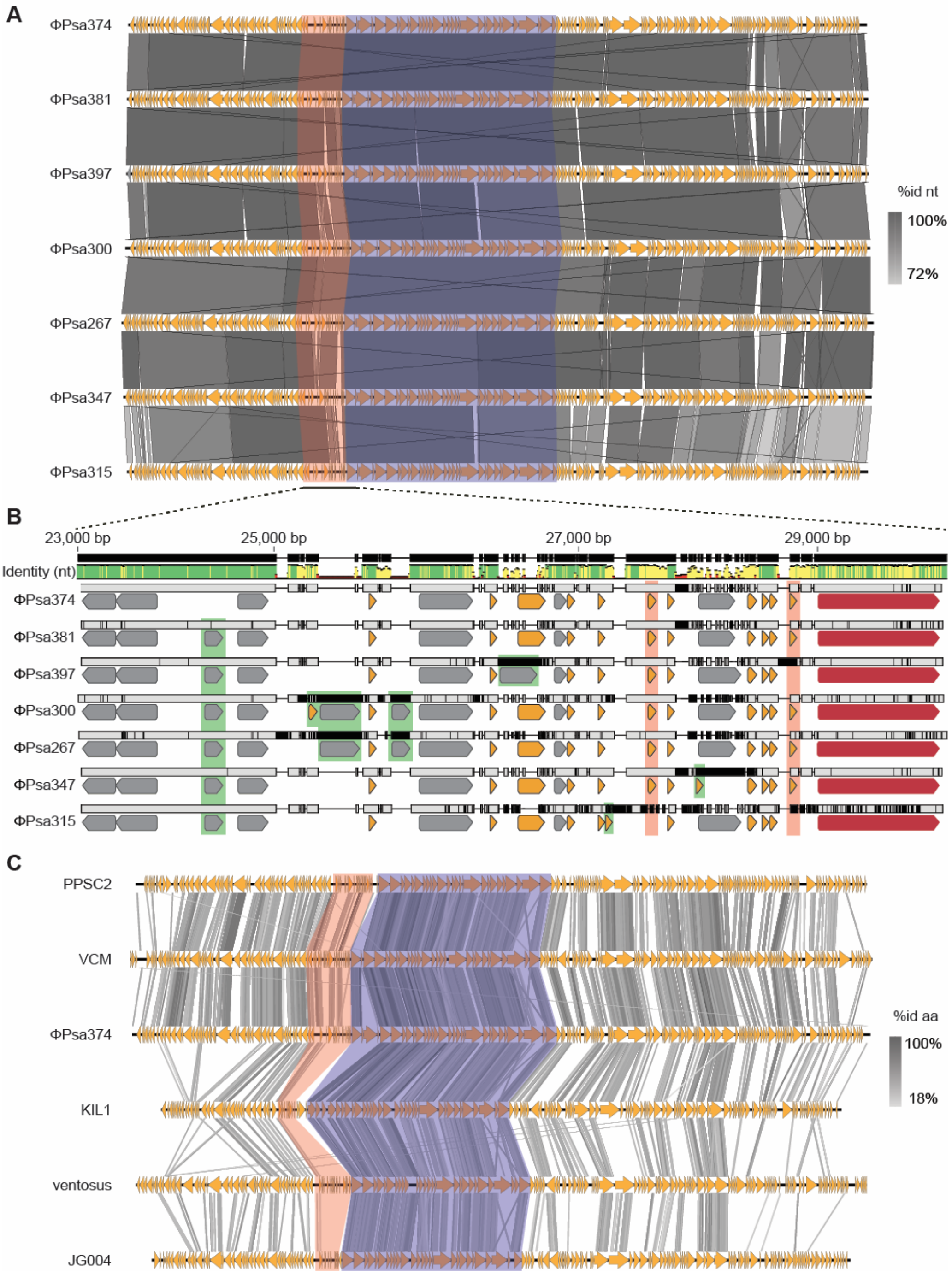

Figure S1. The ΦPsa374-like phages have a high level of sequence conservation. A. blastn whole genome alignment of the ΦPsa374 family tblastx whole genome alignment of phages distantly related to ΦPsa374. **B.** Sequence comparison of the region encoding tRNAs in ΦPsa374-like phages. Hypothetical proteins (grey), tail fiber (pink) and tRNAs (orange). Genetic features gained and lost are shown in green and red boxes, respectively. **C.** tblastx whole genome alignment of phages distantly related to ΦPsa374. In A. and C. conserved tRNAs and structural regions are highlighted in the orange and purple boxes, respectively.

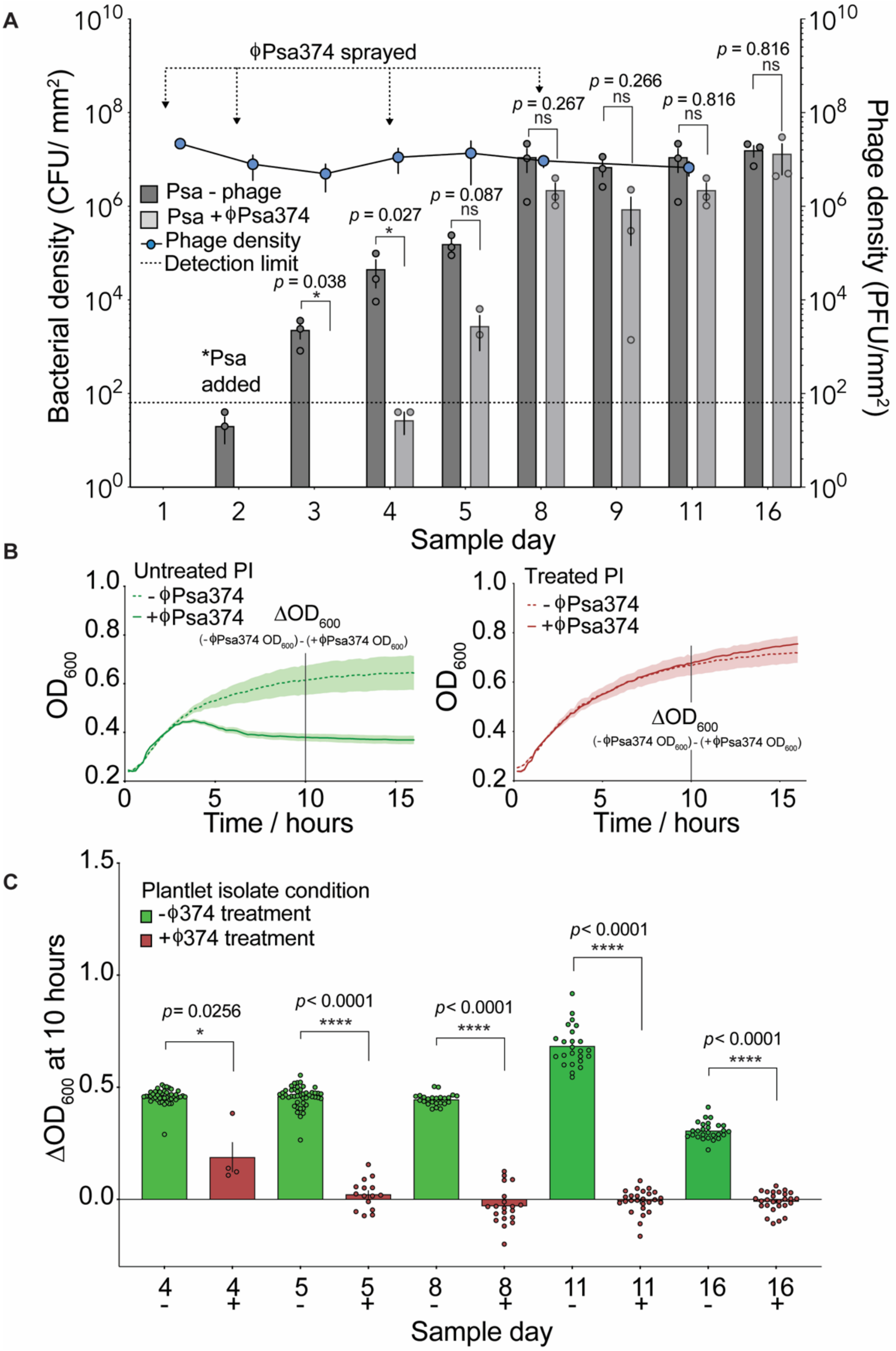

Figure S2. Quadruple ΦPsa374 treatments of *Psa* infected kiwifruit plantlets reduces and delays *Psa* growth but results in phage resistance. A. *Psa* and phage counts from a ϕPsa374 plantlet assay. *Actinidia chinensis* var. *chinensis* plantlets were sprayed with ϕPsa374 on day 1, 2, 4 and 8 and flooded with *Psa* on day 2. Leaf discs were removed from pottles at various time points used to enumerate bacteria (CFU/mm^2; grey bars) and phages (PFU/mm^2; blue circles). Each bar is the average and standard error of the mean (SEM) of

three technical replicates repeated in biological triplicate. Each blue circle is the average and standard deviation of one plating replicate from three biological replicates. **B.** Examples of the growth (OD_{600}) response of *Psa* isolates from untreated and treated kiwifruit plantlets from sample day 16. Untreated plantlet isolates (PI) remain sensitive to ϕPsa374 indicated by a drop in OD_{600} when ϕPsa374 is added to cultures. Treated PI continue to grow in a similar trend to the growth control when ϕPsa374 is added to cultures, indicating the development of resistance to ϕPsa374. **C.** Difference in OD_{600} at 10 hours of growth between the *Psa* PI growth controls and inoculums with ϕPsa374 added from isolates from sample days 4, 5, 8, 11 and 16. A larger difference in OD_{600} indicates that the *Psa* isolates remain sensitive to phage, while little to no difference indicates the development of resistance. *Psa* isolates from plantlets treated with ϕPsa374 were only able to be isolated from sample day 4 (n = 5), 80 % of these isolates show little to no difference in OD_{600} indicating the development of ϕPsa374-resistance. From sample day 5 onwards all isolates show significant resistance to ϕPsa374. Technical and biological replicates are indicated by individual data points. For A. and C. unpaired Welch's t-tests were performed for each of the sample days between the mean of either the bacterial density or ΔOD_{600} between the untreated and ϕPsa374-treated conditions. Significance and *p* values are indicated for each data set.

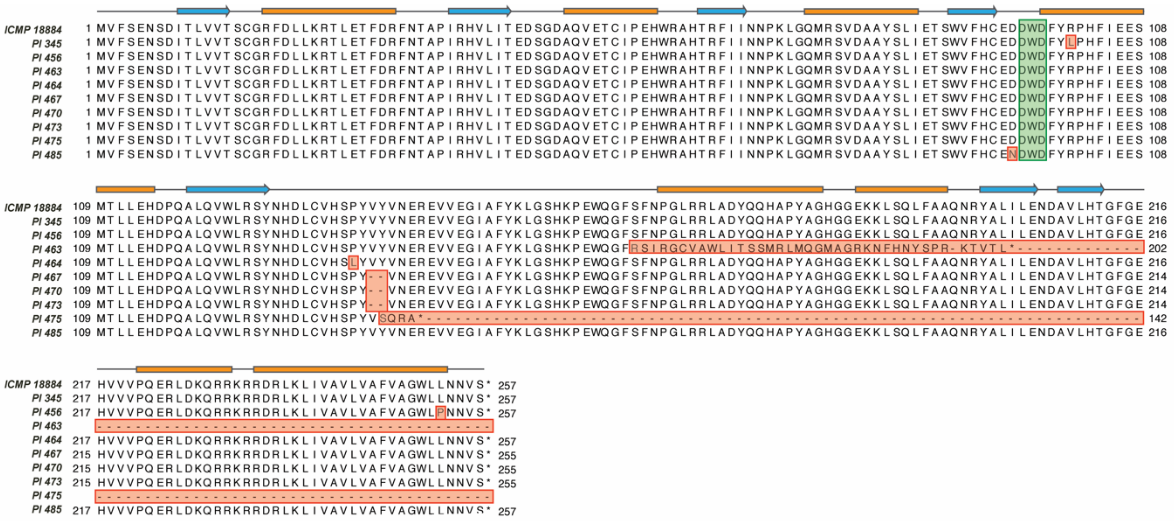

Figure S3. ΦPsa374 treatment of *Psa* infected kiwifruit plantlets causes independent mutations in a glycosyltransferase. Clustal Omega amino acid (aa) alignment of a glycosyltransferase found in the core LPS biosynthetic pathway (locus tag IYO_025560) of *Psa* isolates sampled from kiwifruit plantlets infected with *Psa* and treated with ΦPsa374. Mutations are highlighted in red and the DXD motif typical of this family of glycosyltransferases is highlighted in green. The Phyre2 predicted secondary structure is shown for the glycosyltransferase from *Psa* WT (ICMP 18884) with β-sheets represented by blue arrows and α-helices by orange boxes.

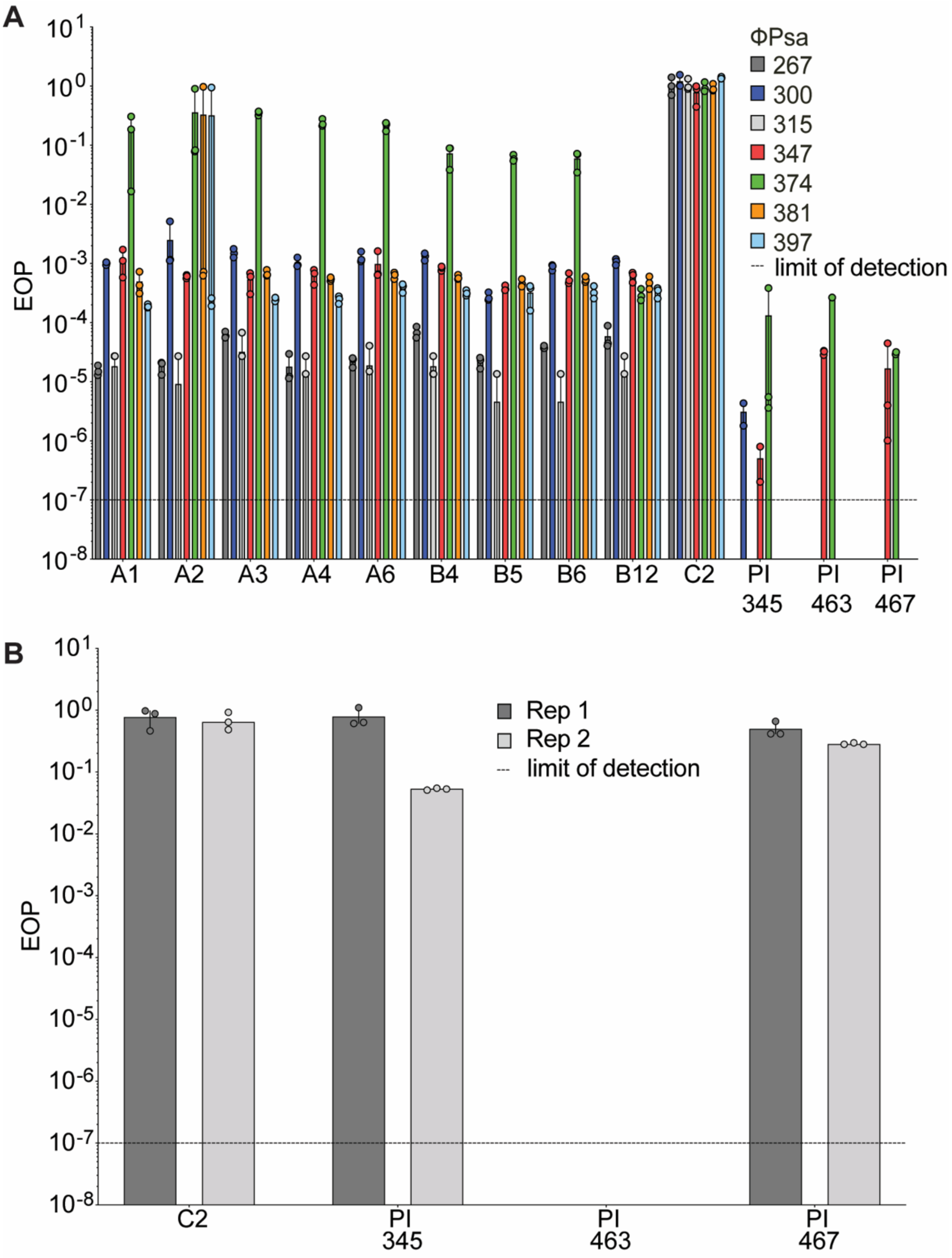

Figure S4. ΦPsa374 and ΦPsa374-like phages utilize LPS as a receptor but can alter binding in response to LPS mutations. A. Raw EOP data for ΦPsa374-like phage heat maps on a panel of LPS-Tn mutants (A1 – B12), a flagellum Tn mutant (C2) and three ΦPsa374-resistant plantlet isolates (PI 345, 463 and 467) presented in Figure 3. **B.** EOP data for individual plaques picked and purified from a *Psa* PI 345 lawn infected with ΦPsa374 against the flagellum Tn mutant (C2) and three ΦPsa374-resistant plantlet isolates (PI 345, 463 and 467). Data for both A. and B. is presented in biological triplicate, each bar represents the mean while points represents each individual replicate. The limit of detection is 10^{-7}, if no data point is present this indicates that plaques were not detected.

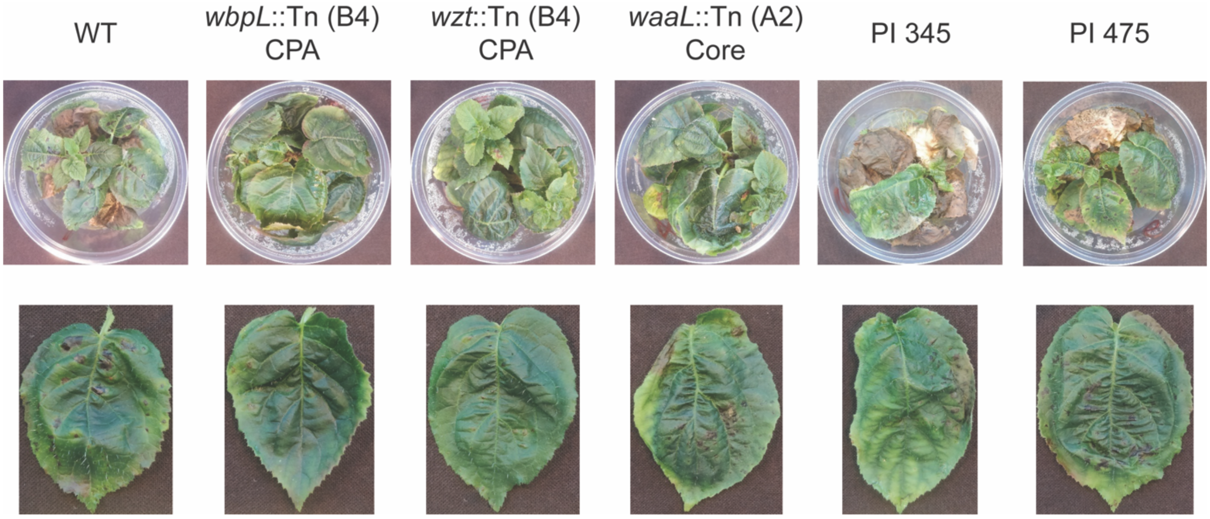

Figure S5. ΦPsa374-resistant *Psa* from *in planta* pathogenicity studies retain pathogenicity, while LPS-Tn mutants do not. Photographs of kiwifruit plantlets and representative leaves from *in planta* studies presented in Figure 4. A significant number of lesions associated with *Psa* disease can be seen in kiwifruit plantlets infected with WT, PI 345, PI 475. Kiwifruit plantlets infected with LPS-Tn mutants do not show any significant disease.

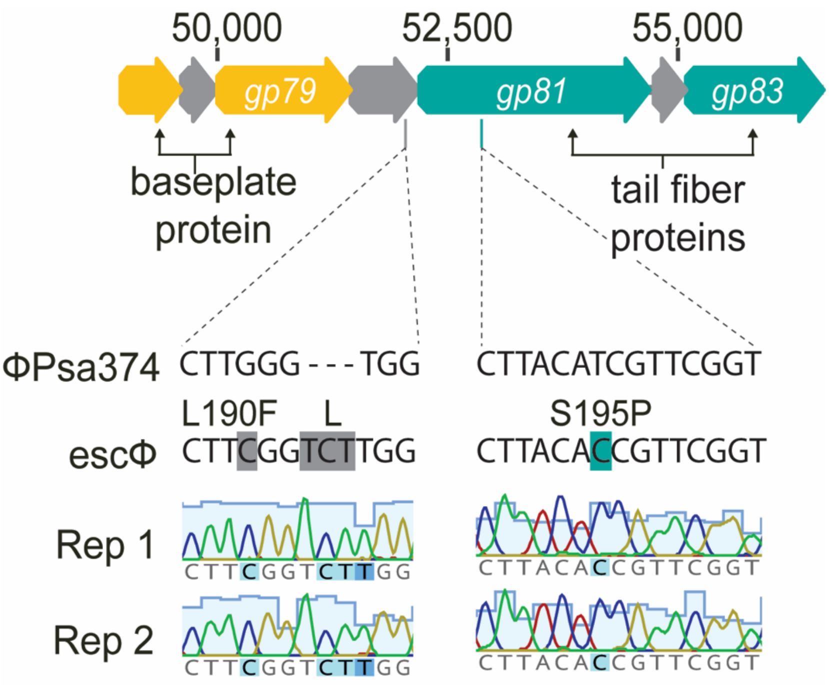

Figure S6. PCR confirmation of mutations in *gp080* and *gp081* in ΦPsa374-escape phages able to overcome ΦPsa374-resistance in *Psa* plantlet isolates. Two plaques (rep 1 and rep 2) found on a lawn of PI 345 infected with escΦ were picked, plaque purified and PCR amplification of genes *gp080* and *gp081* performed. Resultant bands were gel purified and sent for Sanger sequencing. Results for each plaque matched SNPs found in WGS.

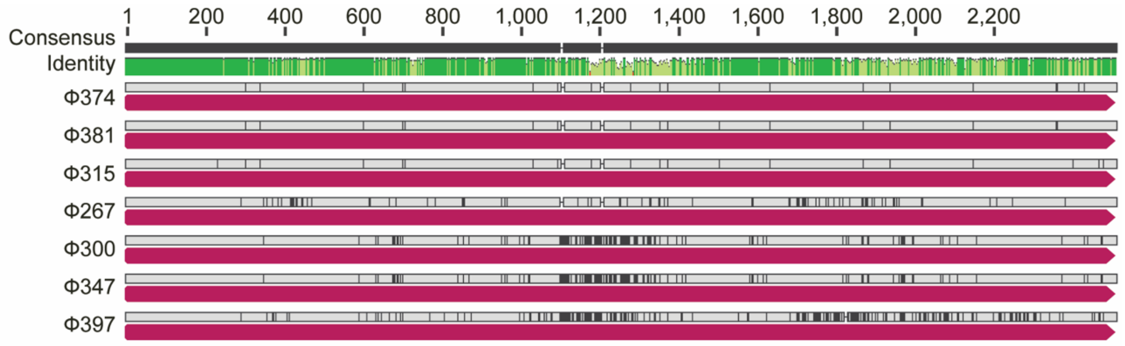

Figure S7. Nucleotide alignment of *gp081* of ΦPsa374-like phages shows conservation at N-termini and variability at C-termini. A multiple nucleotide alignment of *gp081* for ΦPsa374-like phages shows consensus identity conservation in dark green while areas of variability are indicated in light green. For each individual phage gene variation is highlighted in black above each CDS (magenta). Nucleotide alignment was performed in Geneious 6.1.6 using the Clustal Omega algorithm.

Supplementary tables

Table S1. Strains used in this study.

**Strain**	**Notes**	**Reference**
Pseudomonas syringae pv. *actinidiae* ICMP 18884	Aka *Psa* (WT)	[5]
**Transposon mutants**	**Insertion**	**Reference**
A1	gt1::Tn	[6]
A2	*waaL*::Tn	[6]
A3	*wbpL*::Tn	[6]
A4	*rfbD*::Tn	[6]
A6	methyltransferase::Tn	[6]
B4	*wzt*::Tn	[6]
B5	*wbpY*::Tn	[6]
B6	methyltransferase::Tn	[6]
B12	methyltransferase::Tn	[6]
C2	*flgI*::Tn	[6]
**Plantlet isolates**	**Mutation***	**Reference**
PI 406 (Control from plantlets not treated with phage at day 16)	N/A	This study
PI 345	R101L	This study
PI 456	L252P	This study
PI 463	1 bp deletion	This study
PI 464	P135L	This study
PI 467	6 bp deletion	This study
PI 470	6 bp deletion	This study
PI 473	6 bp deletion	This study
PI 475	2 × 1 bp deletion	This study
PI 485	D95N	This study

*Locations of mutations in glycosyltransferase

Table S2. Phages used in this study.

Phages	Reference
Pseudomonas syringae phage ΦPsa374	[7]
Pseudomonas syringae phage ΦPsa267	[7]
Pseudomonas syringae phage ΦPsa300	[7]
Pseudomonas syringae phage ΦPsa315	[7]
Pseudomonas syringae phage ΦPsa347	[7]
Pseudomonas syringae phage ΦPsa381	[7]
Pseudomonas syringae phage ΦPsa397	[7]
Pseudomonas syringae phage ΦPsa21	[7]
Pseudomonas syringae phage ΦPsa374 escape phage	This study

Table S3. ΦPsa374 genome annotation

ORFs	Name	Start (bp)	Stop (bp)	Direction
gp001	hypothetical protein CDS	649	1122	reverse
gp002	hypothetical protein CDS	1318	1458	reverse
gp003	hypothetical protein CDS	1458	1655	reverse
gp004	hypothetical protein CDS	1655	2026	reverse
gp005	hypothetical protein CDS	2023	2145	reverse
gp006	Peptide chain release factor 1 CDS	2145	2537	reverse
gp007	hypothetical protein CDS	2530	2985	reverse
gp008	hypothetical protein CDS	3228	3470	reverse
gp009	hypothetical protein CDS	3636	4028	reverse
gp010	hypothetical protein CDS	4089	4433	reverse
gp011	Tellurium resistance protein TerD CDS	4493	5068	reverse
gp012	Membrane protein TerC CDS	5134	5805	reverse
gp013	hypothetical protein with von Willebrand factor A-like domain TerF CDS	5871	6512	reverse
gp014	hypothetical protein CDS	6528	6782	reverse
gp015	Tellurite resistance protein TelA CDS	6807	7907	reverse
gp016	hypothetical protein CDS	7912	8298	reverse
gp017	hypothetical protein CDS	8300	8803	reverse
gp018	hypothetical protein CDS	8800	9120	reverse
gp019	hypothetical protein CDS	9117	9320	reverse
gp020	hypothetical protein CDS	9329	9544	reverse
gp021	DNA recombination-mediator protein A CDS	9544	10122	reverse
gp022	hypothetical protein CDS	10122	10430	reverse
gp023	hypothetical protein CDS	10423	10584	reverse
gp024	Putative phosphatase	10565	11071	reverse
gp025	hypothetical protein CDS	11068	11190	reverse
gp026	Nicotinamide phosphoribosyltransferase CDS	11236	13011	reverse
gp027	hypothetical protein CDS	13077	13820	reverse
gp028	hypothetical protein CDS	13834	14238	reverse
gp029	Ribose-phosphate pyrophosphokinase CDS	14248	15135	reverse
gp030	hypothetical protein CDS	15198	15536	reverse
gp031	hypothetical protein CDS	15523	15729	reverse
gp032	hypothetical protein CDS	15729	16145	reverse
gp033	RNA ligase, phage-associated CDS	16145	17050	reverse
gp034	hypothetical protein CDS	17053	17274	reverse
gp035	Phosphohydrolase (MutT/nudix family protein) CDS	17274	17843	reverse
gp036	Guanosine-3',5'-bis(diphosphate) 3'-pyrophosphohydrolase CDS	17840	18310	reverse
gp037	hypothetical protein CDS	18368	18847	reverse
gp038	DNA ligase, phage-associated CDS	18858	20015	reverse
gp039	hypothetical protein CDS	20008	20247	reverse
gp040	hypothetical protein CDS	20244	20564	reverse
gp041	hypothetical protein CDS	20564	20797	reverse
gp042	dCMP deaminase CDS	20784	21188	reverse
gp043	hypothetical protein CDS	21379	21585	reverse
gp044	hypothetical protein CDS	21582	21926	reverse
gp045	ATP-dependent Clp protease proteolytic subunit CDS	21995	22606	reverse
gp046	Phosphate starvation-inducible protein PhoH, predicted ATPase CDS	22599	23360	reverse
gp047	hypothetical protein CDS	23402	23746	reverse
gp048	hypothetical protein CDS	23746	24186	reverse
gp049	hypothetical protein CDS	25033	25353	forward
tRNA	tRNA_Val	25821	25891	forward
gp050	hypothetical protein CDS	26137	26697	forward
tRNA	tRNA_Trp	26789	26862	forward
tRNA	tRNA_Arg	26959	27032	forward
gp051	hypothetical protein CDS	27118	27246	forward
tRNA	tRNA_Glu	27255	27328	forward
tRNA	tRNA_Ile	27553	27624	forward
tRNA	tRNA_Asn	27951	28033	forward
tRNA	tRNA_Pro	28155	28225	forward
gp052	hypothetical protein CDS	28462	28614	forward
tRNA	tRNA_Leu	28705	28778	forward
tRNA	tRNA_Cys	28839	28910	forward
tRNA	tRNA_Gly	28920	28992	forward
tRNA	tRNA_Phe	29001	29073	forward
gp053	Phage tail fiber protein CDS – structural protein (MS result)	29286	30563	forward
gp054	hypothetical protein CDS	30579	30842	forward
gp055	Phage tail fiber protein CDS – structural protein (MS result)	30842	32653	forward

ORFs	Name	Start (bp)	Stop (bp)	Direction
gp056	hypothetical protein CDS	32669	32896	forward
gp057	Phage terminase, large subunit CDS	33048	34517	forward
gp058	Putative portal protein CDS – structural protein (MS result)	34529	35986	forward
gp059	Phage capsid and scaffold CDS	35996	36451	forward
gp060	hypothetical protein CDS	36448	37368	forward
gp061	Head decoration protein CDS – structural protein (MS result)	37395	37781	forward
gp062	Phage capsid protein CDS – structural protein (MS result)	37794	38855	forward
gp063	Phage protein CDS – structural protein (MS result)	38906	39385	forward
gp064	hypothetical protein CDS – structural protein (MS result)	39369	39836	forward
gp065	hypothetical protein CDS – structural protein (MS result)	39848	40216	forward
gp066	hypothetical protein CDS – structural protein (MS result)	40213	40782	forward
gp067	Structural protein CDS – structural protein (MS result)	40794	42077	forward
gp068	hypothetical protein CDS – structural protein (MS result)	42112	42636	forward
gp069	hypothetical protein CDS – structural protein (MS result)	42701	43204	forward
gp070	hypothetical protein CDS – structural protein (MS result)	43214	43750	forward
gp071	hypothetical protein CDS	43770	44132	forward
gp072	hypothetical protein CDS	44153	44377	forward
gp073	Tape measure protein CDS – structural protein (MS result)	44390	46747	forward
gp074	hypothetical protein CDS – structural protein (MS result)	46747	47550	forward
gp075	hypothetical protein CDS	47561	47902	forward
gp076	Putative structural protein CDS – structural protein (MS result)	47906	48826	forward
gp077	Putative baseplate protein CDS – structural protein (MS result)	48823	49584	forward
gp078	hypothetical protein CDS – structural protein (MS result)	49593	49958	forward
gp079	Putative baseplate protein CDS – structural protein (MS result)	49961	51418	forward
gp080	Phage protein CDS – structural protein (MS result)	51430	52164	forward
gp081	Phage tail fiber protein CDS – structural protein (MS result)	52176	54653	forward
gp082	hypothetical protein CDS	54703	55071	forward
gp083	Phage tail fiber protein CDS – structural protein (MS result)	55090	56565	forward
gp084	Putative phage-encoded peptidoglycan binding protein CDS	56567	57124	forward
gp085	hypothetical protein CDS	57140	57343	forward
gp086	hypothetical protein CDS	57327	57749	forward
gp087	hypothetical protein CDS	57899	58198	forward
gp088	hypothetical protein CDS	58214	58540	forward
gp089	hypothetical protein CDS	58567	58755	reverse
gp090	hypothetical protein CDS	58748	58891	reverse
gp091	hypothetical protein CDS	58945	59298	reverse
gp092	hypothetical protein CDS	59364	59756	reverse
gp093	Phage protein CDS	60166	60474	forward
gp094	putative DNA/pantothenate metabolism flavoprotein	60471	61103	forward
gp095	hypothetical protein CDS	61100	61315	forward
gp096	hypothetical protein CDS	61312	61545	forward
gp097	hypothetical protein CDS	61547	61762	forward
gp098	hypothetical protein CDS	61759	61953	forward
gp099	hypothetical protein CDS	61950	62270	forward
gp100	hypothetical protein CDS	62331	62948	forward
gp101	hypothetical protein CDS	63352	63564	forward
gp102	hypothetical protein CDS	63548	63676	forward
gp103	hypothetical protein CDS	63673	63855	forward
gp104	hypothetical protein CDS	63855	64178	forward
gp105	T7-like phage primase/helicase protein CDS	64175	66025	forward
gp106	Phage DNA polymerase I CDS	66045	68369	forward
gp107	hypothetical protein CDS	68441	68719	forward
gp108	hypothetical protein CDS	68743	68913	forward
gp109	hypothetical protein CDS	68946	69359	forward
gp110	hypothetical protein CDS	69443	70462	forward
gp111	hypothetical protein CDS	70537	70803	forward
gp112	hypothetical protein CDS	70803	70916	forward
gp113	hypothetical protein CDS	70919	71497	forward
gp114	hypothetical protein CDS	71494	71769	forward
gp115	Phage exonuclease CDS	71810	72844	forward
gp116	hypothetical protein CDS	72844	73068	forward
gp117	Putative HNH endonuclease CDS	73065	73619	forward
gp118	hypothetical protein CDS	73616	73978	forward
gp119	Putative phosphoesterase CDS	73971	74753	forward
gp120	hypothetical protein CDS	74805	75023	forward
gp121	hypothetical protein CDS	75025	75243	forward
gp122	hypothetical protein CDS	75236	75436	forward
gp123	Phage protein CDS	75433	76188	forward
gp124	Putative thymidylate synthase CDS	76265	77206	forward
gp125	hypothetical protein CDS	77199	77537	forward
gp126	Ribonucleotide reductase of class Ia, beta subunit CDS	77546	78607	forward
gp127	Ribonucleotide reductase of class Ia, alpha subunit CDS	78600	80330	forward

ORFs	Name	Start (bp)	Stop (bp)	Direction
gp128	hypothetical protein CDS	80372	80728	forward
gp129	hypothetical protein CDS	80773	81054	forward
gp130	hypothetical protein CDS	81051	81290	forward
gp131	hypothetical protein CDS	81292	81426	forward
gp132	hypothetical protein CDS	81423	81623	forward
gp133	hypothetical protein CDS	81625	81990	forward
gp134	hypothetical protein CDS	81983	82138	forward
gp135	hypothetical protein CDS	82149	82376	forward
gp136	hypothetical protein CDS	82373	82570	forward
gp137	hypothetical protein CDS	82570	82779	forward
gp138	Nucleoside Triphosphate Pyrophosphohydrolase CDS	82790	83203	forward
gp139	Chromosome partition protein smc CDS	83200	83643	forward
gp140	hypothetical protein CDS	83709	83969	forward
gp141	hypothetical protein CDS	83988	84404	forward
gp142	hypothetical protein CDS	84404	84658	forward
gp143	Phage protein CDS	84739	85713	forward
gp144	hypothetical protein CDS	85713	85970	forward
gp145	putative anti-sigma factor	85970	86365	forward
gp146	hypothetical protein CDS	86362	86691	forward
gp147	hypothetical protein CDS	86695	86910	forward
gp148	hypothetical protein CDS	86910	87137	forward
gp149	hypothetical protein CDS	87143	87277	forward
gp150	hypothetical protein CDS	87352	87546	forward
gp151	hypothetical protein CDS	87663	88004	forward
gp152	hypothetical protein CDS	88001	89164	forward
gp153	hypothetical protein CDS	89157	89339	forward
gp154	hypothetical protein CDS	89539	89763	forward
gp155	hypothetical protein CDS	90290	91354	forward
gp156	hypothetical protein CDS	91428	91583	forward
gp157	hypothetical protein CDS	91586	91756	forward
gp158	hypothetical protein CDS	91853	92152	forward
gp159	hypothetical protein CDS	92304	92444	forward
gp160	hypothetical protein CDS	92623	93003	forward
gp161	Phage protein CDS	93068	93766	forward
gp162	hypothetical protein CDS	93822	94163	forward
gp163	hypothetical protein CDS	94272	94541	forward
gp164	hypothetical protein CDS	94568	95071	forward
gp165	hypothetical protein CDS	95144	95719	forward
gp166	hypothetical protein CDS	95719	95892	forward
gp167	hypothetical protein CDS	96061	96504	forward
gp168	hypothetical protein CDS	96569	96787	forward
gp169	hypothetical protein CDS	96856	97056	forward
gp170	hypothetical protein CDS	97056	97322	forward

Table S4. tRNAs encoded by Psa374-like phages.

tRNAs	Psa347-like phage							Closest ortholog		
	374	381	397	300	267	347	315	Organism	Identity (%)	e-value
Ser	-	-	-	+	-	-	-	*Pseudomonas* phage PPSC2	83.3	1.79E-14
Val	+	+	+	+	+	+	+	*Pseudomonas* phage VCM	88.7	1.02E-15
Trp	+	+	+	+	+	+	+	*Pseudomonas* phage VCM	8.7	1.53E-07
Arg	+	+	-	+	+	+	+	*Pseudomonas* phage VCM	91.9	5.01E-20
Glu	+	+	+	+	+	+	+	*Pseudomonas* phage PPSC2	96.0	3.20E-13
Ile	+	+	+	+	+	+	+	no match found		
Arg	-	-	-	-	-	-	+	*Pseudomonas* phage VCM	100	1.45E-26
Asn	+	+	+	+	+	+	-	no match found		
Pro	+	+	+	+	+	+	+	*Pseudomonas* phage VCM	100	1.41E-26
Leu	+	+	+	+	+	+	+	no match found		
Cys	+	+	+	+	+	+	+	*Pseudomonas* phage PPSC2	91.4	4.5E-11
Gly	+	+	+	+	+	+	+	*Pseudomonas* phage ventosus	94.5	3.29E-22
Phe	+	+	-	+	+	+	-	*Pseudomonas* phage PPSC2	78.1	6.35E-06
Total	11	11	9	12	11	11	10			

Table S5. Gene and ORF differences in the structural genome between ΦPsa374 and analogous *Pseudomonas* phages.

Psaϕ374			Orthologous phage structural proteins									
**Locus tag**	**Predicted function**	**Protein size / kDa**	**KIL3** [1]	**Protein size / kDa**	**KIL5** [1]	**Protein size / kDa**	**PaP1** [2]	**Protein size / kDa**	**PAK_P3** [3]	**Protein size / kDa**	**PaoP5** [4]	**Protein size / kDa**
gp053	Phage tail fiber protein	44.7	-	-	-	-	-	-	-	-	-	-
gp054	Hypothetical protein	8.8	*ORF41* (vB_PsyM_KIL3_0046)	9.1	-	-	-	-	-	-	-	-
gp055	Phage tail fiber protein	63.0	*ORF42* (vB_PsyM_KIL3_0047)	30.58	*ORF42* (vB_PsyM_KIL5_0051)	30.62	-	-	-	-	-	-
gp056	Hypothetical protein	7.6	*ORF43* (vB_PsyM_KIL3_0048)	4.2	*ORF43* (vB_PsyM_KIL5_0052)	4.2	-	-	-	-	-	-
gp057	Phage terminase, large subunit	55.9	*ORF44* (vB_PsyM_KIL3_0049)	51.97	*ORF44* (vB_PsyM_KIL5_0053)	52.22	*gp046*	57.1	*gp001*	*53.6*	*gp052*	57
-	-	-	*ORF45* (vB_PsyM_KIL3_0050)	21.91	*ORF45* (vB_PsyM_KIL5_0054)	20.7	-	-	-	-	-	-
gp058	Putative portal protein	54.5	*ORF46* (vB_PsyM_KIL3_0051)	54.78	*ORF46* (vB_PsyM_KIL5_0055)	54.76	*gp047*	54.23	*gp002*	56.3	*gp053*	54.2
gp059	Phage capsid and scaffold	16.8	*ORF47* (vB_PsyM_KIL3_0052) Putative methyltransferase	17.1	*ORF47* (vB_PsyM_KIL5_0056) Putative phage protease XkdF	18.45	*gp048* DNA-specific methyltransferase	17.2	*gp003* Putative methyltransferase	15.2	*gp054*	17.1
gp060	Hypothetical protein	33.2	*ORF48* (vB_PsyM_KIL3_0053)	35.57	*ORF48* (vB_PsyM_KIL5_0057)	35.59	*gp049*	33.1	*gp004*	31.8	*gp055*	31
gp061	Head decoration protein	13.7	*ORF49* (vB_PsyM_KIL3_0054)	14.57	*ORF49* (vB_PsyM_KIL5_0058)	14.57	*gp050*	14.87	*gp005*	13.5	*gp056*	15
gp062	Phage capsid protein	39.5	*ORF50* (vB_PsyM_KIL3_0055)	39.84	ORF50 (vB_PsyM_KIL5_0059)	39.87	*gp051*	39.38	*gp006*	40.2	*gp057*	39

Psaϕ374			Orthologous phage structural proteins									
**Locus tag**	**Predicted function**	**Protein size / kDa**	**KIL3** [1]	**Protein size / kDa**	**KIL5** [1]	**Protein size / kDa**	**PaP1** [2]	**Protein size / kDa**	**PAK_P3** [3]	**Protein size / kDa**	**PaoP5** [4]	**Protein size / kDa**
gp063	Phage protein	18.0	*ORF51* (vB_PsyM_KIL3_0056)	23.06	*ORF51* (vB_PsyM_KIL5_0060)	22.6	*gp052*	18.1	*gp007*	18.6	*gp059*	18.1
gp064	Hypothetical protein	17.1	*ORF52* (vB_PsyM_KIL3_0057)	24.06	*ORF52* (vB_PsyM_KIL5_0061) Putative RNA polymerase	17.3	*gp053* *Putative RNA polymerase*	10.5	*gp008*	17.5	*gp060* Putative RNA polymerase	8.8
gp065	Hypothetical protein	13.9	*ORF53* (vB_PsyM_KIL3_0058)	13.97	*ORF53* (vB_PsyM_KIL5_0062)	13.97	*gp054*	14.3	*gp009*	14.3	*gp061*	14.3
-	-	-	*ORF54* (vB_PsyM_KIL3_0059)	28.64	-	-	-	-	-	-	-	-
gp066	Hypothetical protein	21.7	*ORF55* (vB_PsyM_KIL3_0060)	23.06	*ORF54* (vB_PsyM_KIL5_0063)	22.26	*gp055*	21.29	*gp010*	23.4	*gp062*	21.3
-	Hypothetical protein	-	*ORF56* (vB_PsyM_KIL3_0061)	25.9	*ORF55* (vB_PsyM_KIL5_0064)	8.6	-	-	-	-	-	-
gp067	Structural protein	45.8	*ORF57* (vB_PsyM_KIL3_0062)	45.76	*ORF56* (vB_PsyM_KIL5_0065)	45.76	*gp056*	46.37	*gp011*	47.9	*gp063*	47
-	-	-	*ORF58* (vB_PsyM_KIL3_0063) Putative endonuclease	26.7	*ORF57* (vB_PsyM_KIL5_0066) Putative endonuclease	11.4	-	-	-	-	-	-
-	-	-	-	-	*ORF58* (vB_PsyM_KIL5_0067) Hypothetical protein	4.0	-	-	-	-	-	-
-	-	-	-	-	*ORF59* (vB_PsyM_KIL5_0068) Putative homing endonuclease	7.3	-	-	-	-	-	-

Psaϕ374			Orthologous phage structural proteins									
**Locus tag**	**Predicted function**	**Protein size / kDa**	**KIL3** [1]	**Protein size / kDa**	**KIL5** [1]	**Protein size / kDa**	**PaP1** [2]	**Protein size / kDa**	**PAK_P3** [3]	**Protein size / kDa**	**PaoP5** [4]	**Protein size / kDa**
gp068	Hypothetical protein	18.7	*ORF59* (vB_PsyM_KIL3_0064)	19.92	*ORF60* (vB_PsyM_KIL5_0069) Putative structural protein	18.81	*gp057* Putative structural protein	18.97	*gp012*	18.8	*gp064*	19
gp069	Hypothetical protein	18.0	*ORF60* (vB_PsyM_KIL3_0065)	18.0	*ORF61* (vB_PsyM_KIL5_0070)	22.93	*gp058*	18.4	*gp013*	18.3	gp065	13.5
gp070	Hypothetical protein	19.0	*ORF61* (vB_PsyM_KIL3_0066)	19.14	*ORF62* (vB_PsyM_KIL5_0071)	19.15	*gp059* Putative structural protein	17.7	*gp014*	18.7	gp066	17.7
-	-	-	*ORF62* (vB_PsyM_KIL3_0067) Putative homing endonuclease	12.9	*ORF63* (vB_PsyM_KIL5_0072) Hypothetical protein	7.8	-	-	-	-	-	-
gp071	Hypothetical protein	12.9	*ORF63* (vB_PsyM_KIL3_0068)	12.9	*ORF64* (vB_PsyM_KIL5_0073)	13.01	*gp060* Putative structural protein	13.6	*gp015*	13.9	gp067	13.5
gp072	Hypothetical protein	8.7	*ORF64* (vB_PsyM_KIL3_0069)	11.1	ORF65 (vB_PsyM_KIL5_0074)	11.09	*gp161*	9.9	*gp016*	8.7	-	-
gp073	Tape measure protein	84.4	*ORF65* (vB_PsyM_KIL3_0070)	87.69	*ORF66* (vB_PsyM_KIL5_0075)	85.3	*gp061*	85.87	*gp017*	84.8	*gp068*	85.4
gp074	Hypothetical protein	29.4	*ORF66* (vB_PsyM_KIL3_0071)	29.54	*ORF67* (vB_PsyM_KIL5_0076)	29.86	*gp062*	28.5	*gp018*	27.9	*gp069*	28.5
gp075	Hypothetical protein	13.5	*ORF67* (vB_PsyM_KIL3_0072)	13.4	*ORF68* (vB_PsyM_KIL5_0077)	13.62	*gp063*	13.9	*gp019*	14.6	*gp070*	13.9
gp076	Putative structural protein	34.1	*ORF68* (vB_PsyM_KIL3_0073)	35.11	*ORF69* (vB_PsyM_KIL5_0078)	35.11	*gp064*	33.8	*gp020*	32.6	*gp071*	17.6

Psaϕ374			Orthologous phage structural proteins									
**Locus tag**	**Predicted function**	**Protein size / kDa**	**KIL3** [1]	**Protein size / kDa**	**KIL5** [1]	**Protein size / kDa**	**PaP1** [2]	**Protein size / kDa**	**PAK_P3** [3]	**Protein size / kDa**	**PaoP5** [4]	**Protein size / kDa**
gp077	Putative baseplate protein	27.4	*ORF69* (vB_PsyM_KIL3_0074)	27.97	*ORF70* (vB_PsyM_KIL5_0079)	27.98	*gp065*	26.7	*gp021*	27.2	*gp072*	26.7
gp078	Hypothetical protein	13.4	*ORF70* (vB_PsyM_KIL3_0075) Tail lysozyme	13.3	*ORF71* (vB_PsyM_KIL5_0080) Tail lysozyme	16.41	*gp066*	14.2	*gp022*	14.1	*gp073*	14.2
gp079	Putative baseplate protein	51.7	*ORF71* (vB_PsyM_KIL3_0076)	53.46	ORF72 (vB_PsyM_KIL5_0081)	53.43	*gp067*	52.4	*gp023*	53.3	*gp074*	50
gp080	Phage protein	26.6	*ORF72* (vB_PsyM_KIL3_0077)	26.79	*ORF73* (vB_PsyM_KIL5_0082)	26.79	*gp068*	26.7	*gp024*	28.1	*gp075*	28
gp081	Phage tail fiber protein	85.5	*ORF73* (vB_PsyM_KIL3_0078)	72	*ORF74* (vB_PsyM_KIL5_0083)	72.03	*gp069*	69.79	*gp025*	70.2	*gp076*	74
gp082	Hypothetical protein	13.6	*ORF74* (vB_PsyM_KIL3_0079)	15.6	*ORF75* (vB_PsyM_KIL5_0084)	16.04	*gp070*	13.6	*gp026*	14.7	*gp077*	14.5
gp083	Phage tail fiber protein	51.7	*ORF75* (vB_PsyM_KIL3_0080)	51.18	*ORF76* (vB_PsyM_KIL5_0085)	51.17	*gp071*	53.09	*gp027*	52.9	*gp078*	55
gp084	Endolysin	20.6	*ORF76* (vB_PsyM_KIL3_0081)	20.84	*ORF77* (vB_PsyM_KIL5_0086)	20.84	*gp072*	20.9	*gp028*	20.8	*gp079*	20.8

*Cells shaded in orange indicate proteins identified by ESI-MS/MS, all protein sizes are predicted unless identified in ESI-MS/MS, dashes (-) indicate gene not present, annotations different to PsaΦ374 or orthologous phage are in red text.

Table S6. SNPs found in the gene IYO_025560 of *Psa* isolates sampled from ΦPsa374-treated kiwifruit plantlets infected with *Psa* ICMP 18884.

**Isolate**	**Sample day**	**Bio /tech. rep. no.**	**Mutation location**	**CDS position (bp)**	**Codon change**	**Protein effect**
PI 345	11	3, 3	5,648,985	302	CGT → CTT	Substitution
PI 456	16	1, 1	5,648,532	755	CTC → CCC	Substitution
PI 463	16	1, 3	5,648,801	486	AAC → A-C	Frame shift
PI 464	16	1, 3	5,648,883	404	CCC → CTC	Substitution
PI 467	16	2, 1	5,648,877	405	CCC, TAT, GTC → CCC	Deletion
PI 470	16	2, 1	5,648,877	405	CCC, TAT, GTC → CCC	Deletion
PI 473	16	2, 2	5,648,877	405	CCC, TAT, GTC → CCC	Deletion
PI 475	16	2, 2	5,648,875	409	TAT → -A-	Frame shift
PI 485	16	3, 1	5,649,004	283	GAT → AAT	Substitution

Supplementary References

1. Rombouts, S. *et al.* Characterization of novel bacteriophages for biocontrol of bacterial blight in leek caused by *Pseudomonas syringae* pv. *porri*. *Front. Microbiol.* **7**, 1–15 (2016).

2. Lu, S. *et al.* Unlocking the mystery of the hard-to-sequence phage genome: PaP1 methylome and bacterial immunity. *BMC Genomics* **15**, 803 (2014).

3. Henry, M. *et al.* The search for therapeutic bacteriophages uncovers one new subfamily and two new genera of *Pseudomonas*-infecting myoviridae. *PLoS One* **10**, 1–16 (2015).

4. Shen, M. *et al.* Characterization and Comparative Genomic Analyses of *Pseudomonas aeruginosa* Phage PaoP5: New Members Assigned to PAK-P1-like Viruses. *Sci. Rep.* **6**, 1–6 (2016).

5. Templeton, M. D., Warren, B. A., Andersen, M. T., Rikkerink, E. H. A. & Fineran, P. C. Complete DNA Sequence of *Pseudomonas syringae* pv. *actinidiae*, the Causal Agent of Kiwifruit Canker Disease. *Genome Announc.* **3**, e01054-15 (2015).

6. Mesarich, C. H. *et al.* Transposon insertion libraries for the characterization of mutants from the kiwifruit pathogen *Pseudomonas syringae* pv. *actinidiae*. *PLoS One* **12**, 1–21 (2017).

7. Frampton, R. A. *et al.* Identification of Bacteriophages for Biocontrol of the Kiwifruit Canker Phytopathogen *Pseudomonas syringae* pv. *actinidiae*. *Appl. Environ. Microbiol.* **80**, 2216–2228 (2014).
